# Moving beyond PARP Inhibition: Current State and Future Perspectives in Breast Cancer

**DOI:** 10.3390/ijms22157884

**Published:** 2021-07-23

**Authors:** Michela Palleschi, Gianluca Tedaldi, Marianna Sirico, Alessandra Virga, Paola Ulivi, Ugo De Giorgi

**Affiliations:** 1 Department of Medical Oncology, IRCCS Istituto Romagnolo per lo Studio dei Tumori (IRST) “Dino Amadori”, 47014 Meldola, Italy; michela.palleschi@irst.emr.it (M.P.); marianna.sirico@irst.emr.it (M.S.); ugo.degiorgi@irst.emr.it (U.D.G.); 2 Biosciences Laboratory, IRCCS Istituto Romagnolo per lo Studio dei Tumori (IRST) “Dino Amadori”, 47014 Meldola, Italy; alessandra.virga@irst.emr.it (A.V.); paola.ulivi@irst.emr.it (P.U.)

**Keywords:** PARP inhibitors, breast cancer, PARP inhibitor resistance

## Abstract

Breast cancer is the most frequent and lethal tumor in women and finding the best therapeutic strategy for each patient is an important challenge. PARP inhibitors (PARPis) are the first, clinically approved drugs designed to exploit synthetic lethality in tumors harboring *BRCA1/2* mutations. Recent evidence indicates that PARPis have the potential to be used both in monotherapy and combination strategies in breast cancer treatment. In this review, we show the mechanism of action of PARPis and discuss the latest clinical applications in different breast cancer treatment settings, including the use as neoadjuvant and adjuvant approaches. Furthermore, as a class, PARPis show many similarities but also certain critical differences which can have essential clinical implications. Finally, we report the current knowledge about the resistance mechanisms to PARPis. A systematic PubMed search, using the entry terms “PARP inhibitors” and “breast cancer”, was performed to identify all published clinical trials (Phase I-II-III) and ongoing trials (ClinicalTrials.gov), that have been reported and discussed in this review.

## 1. Introduction

Breast cancer (BC) is the most commonly diagnosed cancer and the leading cause of cancer death in women [1]. One of the several risk factors for BC development is genetic predisposition, mainly linked to mutations in *BRCA1* and *BRCA2* genes [2]. In the early years of the current millennium, the evidence that *BRCA1/2*-mutant cells could be sensitive to PARP inhibitors (PARPis) emerged and paved the way for new therapeutic opportunities in different tumors, including BC [3,4,5,6].

Olaparib (Lynparza^®^) was the first PARPi approved by the Food and Drug Administration (FDA) in 2014 for the treatment of patients with advanced ovarian cancer and germline or somatic mutations in *BRCA1/2* genes who have been previously treated with three or more lines of chemotherapy [7]. Specific approval for advanced epithelial ovarian, fallopian tube or primary peritoneal cancer maintenance treatments occurred after few years, based on SOLO-1 clinical trial [8]. Selected patients for this treatment are in complete or partial response to first-line platinum-based therapy and carriers of germline or somatic *BRCA1/2* mutations [9]. In 2020 the use of olaparib in combination with bevacizumab was approved as a maintenance treatment for patients with advanced ovarian cancer [10].

Olaparib efficacy was demonstrated also in other tumors, such as pancreatic, prostate and breast cancers [11]. In particular, olaparib was approved by the FDA in 2019 as a maintenance treatment for patients with germline *BRCA1/2* mutations and metastatic pancreatic adenocarcinoma [12], after the demonstration of its efficacy in the multi-center trial POLO [13]. In addition, in 2018, olaparib has been authorized also in the treatment of metastatic BC, germline *BRCA1/2*-mutated and HER2-negative, based on OlympiAD clinical trial [14]. Finally, in 2020, the FDA approved the use of olaparib also in metastatic castration-resistant prostate cancers (mCRPC) with germline or somatic *BRCA1/2* mutations previously treated with Enzalutamide and/or Abiraterone, as demonstrated in the PROfound clinical trial [15,16].

Since 2016, other PARPis were authorized for the treatment of ovarian, prostate and breast cancers. In 2016, FDA approved rucaparib (Rubraca^®^) for the treatment of *BRCA1/2*-mutated patients with advanced ovarian carcinoma [17]. TRITON2 and TRITON3 clinical trials evaluated the efficacy of rucaparib also in mCRPC patients with germline or somatic *BRCA1/2* mutations [18].

Moreover, niraparib (Zejula^®^) has recently been approved for maintenance treatment of ovarian cancer patients based on the PRIMA clinical trial [19,20]. Finally, talazoparib (Talzenna^®^) has been authorized in 2018 for the treatment of BCs with germline *BRCA1/2* mutations based on the EMBRACA clinical trial [21].

Finally, several phase-II and phase-III trials are in progress focusing on the efficacy of new PARPis in the treatment of advanced ovarian cancer, advanced prostate cancer and non-small cell lung cancer (NSCLC) [22]. Current clinical trials on olaparib for the treatment of triple-negative breast cancer (TNBC) are evaluating also the PARPi efficacy in patients who carry mutations in other homologous recombination genes such as *PALB2*, *ATM*, *RAD51* [23].

## 2. DNA Damage Repair and Mechanism of Action of PARPis

DNA instability is an important characteristic of carcinogenesis. Endogenous and exogenous factors are responsible for DNA damage, such as chemical and physical agents including ROS or ultraviolet radiations [24]. Tumor takes advantage of these damaging agents, bypassing cellular repair mechanisms and upsetting correct signaling networks. In addition, chemotherapy and radiation therapy, despite their benefits, can be considered among DNA mutational agents [25].

Once the DNA damage has occurred, cells activate different repair systems. Some of them act on single base mutation, such as base excision repair (BER), nucleotide excision repair (NER) and DNA mismatch repair (MMR) [26,27,28]. Regarding double-strand breaks (DSB), the damage repair is mediated by nonhomologous end-joining (NHEJ) and homologous recombination repair (HRR) systems [29]. The first DNA repair system directly binds DNA breaks but it can introduce alterations in the sequence. On the contrary, in the second case, there is the necessity to have a strand to guide the repair and replicate the sequence correctly. Schwart et al. demonstrated that downregulation of NHEJ and HRR key components generated fragile sites on DNA, proving the two systems are complementary and essential to maintain chromosome stability [30].

**Figure 1:**
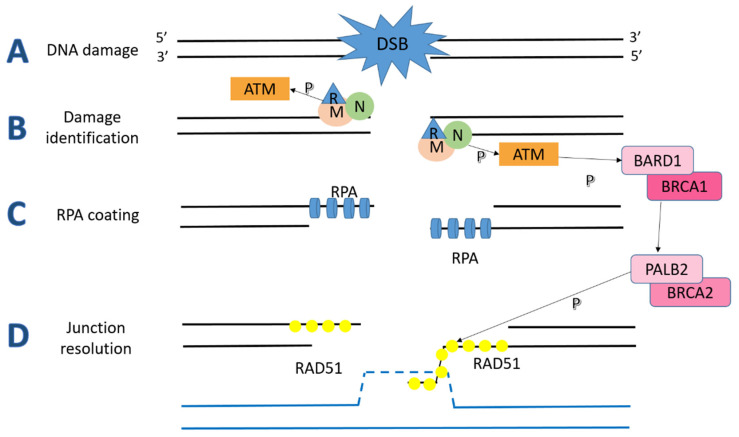
When a DSB occurs in the DNA (**A**), the MRN complex recognizes the damage (**B**) and starts 5′ strand degradation to get a 3′ ssDNA free to be coated by RPA and ATM is activated by phosphorylation (**C**). The interaction of RPA and RAD51 repairs the damage with the support of BRCA1-BARD1 and BRCA2-PALB2 complexes (**D**).

The HRR process consists of three main phases: damage recognition, strand preparation and junction resolution (Figure 1). The damage identification is the result of the activity of the MRN complex, composed of Mre11, Rad50, and Nbs1 proteins, that starts the degradation of the 5′ strand, in collaboration with CtBP-interacting protein (CtIP) nuclease, recruits and activates the upstream ATM kinase [31,32,33]. Subsequently, ATM phosphorylates the BRCA1 protein, which is involved in the third process phase [34,35].

The second phase involves Replication Protein A (RPA), a heterotrimeric protein that binds to just generated 3′ single-strand DNA (ssDNA) [36]. RPA tightly cooperates with RAD51 protein, a DNA-dependent ATPase, binding ssDNA within a gap and pairs linear dsDNA with a small circular ssDNA (called intermediate joint). Meanwhile, RPA promotes the RAD51-DNA link by removing secondary structures and stabilizing the intermediate joint through the non-complementary DNA strand sequestration [37,38,39,40].

In the third phase, RAD51 recombinase activity is enhanced by the activated BRCA1-BARD1 complex, which interacts with both ssDNA and RAD51 [41,42]. The coiled-coil domain of BRCA1 binds the PALB2-BRCA2 complex, which stabilizes RAD51 nucleofilament and promotes the interaction between RAD51 and RPA [43,44]. CDK12, cyclinD1, CHK1 and many other players cooperate to complete the structural mechanism of the HRR pathway [45,46].

Mutations in genes coding the HRR system components can alter the process and generate a homologous recombination deficiency (HRD). The HRD is a frequent driver of tumorigenesis in many tumors, in particular ovarian, pancreatic, prostate and breast cancers [2,47,48,49,50].

However, the HRD signature can be exploited for personalized medicine since it is predictive of the sensitivity to targeted therapy with inhibitors of the poly ADP-ribose polymerase (PARP) enzyme, as well as DNA damaging reagents [5,47,51,52,53].

**Figure 2:**
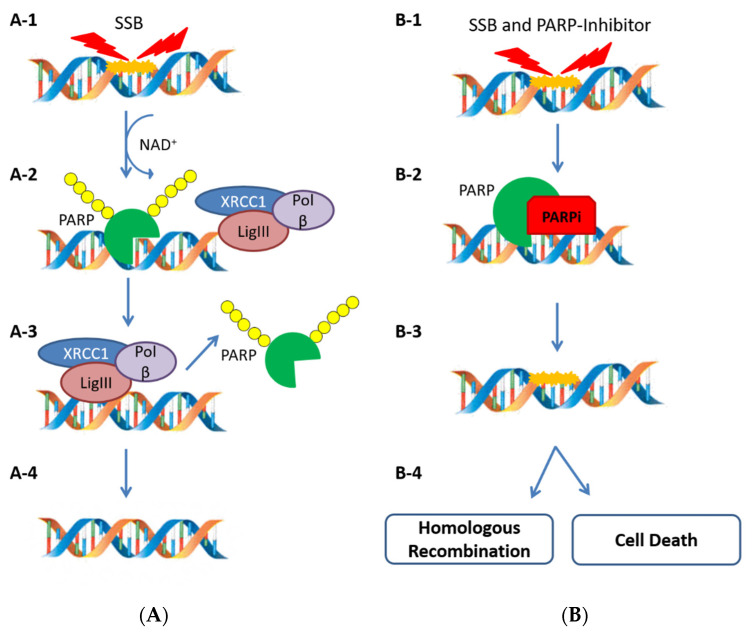
When an SSB occurs (**A-1**), PARP-1 recruits scaffold proteins and forms an ADP-ribose chain on itself (PARylation) using NAD+ (**A-2**). The PARylation also promotes PARP-1 dissociation and leads scaffold proteins to repair the SSB (**A-3** and **A-4**). When an SSB occurs in presence of a PARPi (**B-1**), the PARPi binds the NAD+ binding site in a competitive way (**B-2**). Since the SSB cannot be repaired by PARP enzyme, the HRR system can repair the damage but the coexistence of HRD prevents the repair and induces cell death (**B-3** and **B-4**).

PARP is a family of 17 proteins essential in the BER system, involved in DNA single-strand break (SSB) repair [54]. In particular, PARP-1, PARP-2, and PARP-3 are primarily involved in DNA damage repair, otherwise PARP-5a and PARP-5b act in the regulation of mitosis and telomere maintenance for the conservation of chromosome stability [55]. As DNA damage sensors, PARP proteins bind DNA and start the synthesis of a poly (ADP-Ribose) chain (PARylation), which acts as a recruitment signal for other scaffold and regulatory proteins, such as DNA Ligase III (LigIII), DNA polymerase beta (polβ) and X-ray Cross Complementing Protein 1 (XRCC1) to repair the DNA damage (Figure 2A) [56].

The use of PARPis generates an impairment of the BER system and the inability to repair SSBs. In this situation, SSBs are converted into DSBs and the HRR system becomes essential to repair the damage [57,58].

This process can be exploited in cancer therapy through the mechanism of synthetic lethality. Indeed, if the cell is characterized by an HRD, caused by a mutation in *BRCA1/2* or other HRR genes, the treatment with a PARPi generates a genetic instability that leads to cell death (Figure 2B) [3]. Substantially, the PARPi treatment is a targeted therapy that selects HRD cancer cells and brings exclusively them to death [59,60].

## 3. Clinical Development of PARPis as a Single Agent in Breast Cancer Treatment

### 3.1. Advanced and/or Metastatic Breast Cancer

The first phase-I trial evaluating olaparib in metastatic breast cancer (mBC) was reported in 2009 by Fong et al. [53]. This trial enrolled 60 patients with refractory disease to standard therapies or those for whom there were no suitable or effective standard treatments. They were treated using a dose-escalation strategy ranging from 10 mg per day to 600 mg twice per day. Of these patients, nine had mBC and three of them carried a *BRCA1/2* mutation. One of the latter three patients showed a complete response according to the RECIST classification, lasting for 60 weeks. Mainly grade I and II adverse events were reported and included digestive (anorexia, nausea, vomiting) and hematological (anemia, thrombocytopenia) toxicities. These initial results led to the completion of three phase-II trials evaluating olaparib in BC. The first, published in 2010, by Tutt et al. [52], recruited 54 patients with locally advanced or mBC carrying a *BRCA1/2* mutation. They were divided into two cohorts of 27 patients; the first cohort received 400 mg olaparib twice daily (which was found to be the maximum tolerated dose in the phase-I trial) and the second was treated with 100 mg olaparib twice daily (minimum pharmacodynamically effective dose as identified in the phase-I trial). The main goal of this study was to determine the objective response rate (ORR). The 41% (11 out of 27) in the first cohort responded to olaparib therapy, with one patient in complete response and ten in partial response, while the 22% (6 out of 27) in the second cohort, did not exhibit complete response. Again, mainly grade I and II adverse effects, similar to those experienced in the phase-I trial, were reported. The second study by Gelmon et al. [61], treated patients with 400 mg olaparib twice daily but unfortunately could not determine a conclusive ORR after treatment. Finally, Kaufman et al. [62] reported an ORR of 12.9% in their cohort of 62 heavily pretreated patients, all bearing *BRCA1/2*-mutated mBC. In 2017, Robson et al. [14] reported the first randomized phase-III trial comparing olaparib with standard chemotherapy in patients with HER2-negative mBC carrying a *BRCA1/2* germline mutation with resistance to hormone treatment and having received no more than two lines of chemotherapy for the treatment of their mBC. A total of 302 patients were randomized (2:1), 205 received olaparib at a dose of 300 mg twice daily (new dosage form in film-coated tablets) and 97 received clinician’s choice chemotherapy (vinorelbine, eribulin or capecitabine). The study met its primary endpoint of a statistically significant increase in progression-free survival (PFS); however, there was no significant difference in overall survival (OS) between the two groups. The most frequently observed hematological adverse reaction was anemia [63]. These results led to the approval of olaparib by the FDA and the European Medicines Agency (EMA), for locally advanced or HER2-negative mBCs, as a monotherapy.

In addition to the inhibition of catalytic activity common to all PARPis, talazoparib has exhibited in vitro “trapping” of the PARP complex at the most important breakage site. In 2017, De Bono et al. [64] published the results of the first phase-I trial evaluating talazoparib in the treatment of advanced solid tumors. The trial consisted of two phases: a dose-escalation phase in which patients received talazoparib at a dose between 0.025 mg/day and 1.1 mg/day and an expansion phase comprising 71 patients receiving talazoparib at the recommended dose of 1 mg/day. Significant PARP inhibition was observed for a dose of 0.60 mg/day, with hematological toxicity reported to be the most frequent adverse effect, although reversible with the suspension of treatment and reduction of doses. The reported ORR was 50% and included one patient with complete response. Further to this, Turner et al. [65] reported the results of the ABRAZO trial: a phase-II trial evaluating talazoparib at a dose of 1 mg/day in patients with locally advanced or mBC and with a *BRCA1/2* germline mutation. In this trial, the population under investigation consisted of 2 cohorts of individuals: the first cohort included patients who had previously been treated with platinum salts while the second cohort enrolled patients without prior exposure to platinum. The ORR for cohort 1 was 21% and 37% for cohort 2, and the most common adverse reaction was anemia. In 2018, Litton et al. [66] conducted the first randomized phase-III trial comparing talazoparib with chemotherapy (capecitabine, eribulin, gemcitabine or vinorelbine) in germline *BRCA1/2*-mutated HER2-negative advanced BC. All patients randomized to talazoparib started at 1 mg once daily. This trial met its primary endpoint of a statistically significant increase in PFS from 5.6 months to 8.6 months and the ORR was found to be twice as high in patients treated with talazoparib (62.6% vs. 27.2%). The most frequent adverse event was haematological toxicity for all talazoparib treatment arms and approximately 50% of patients in the chemotherapy arm. The other predominantly reported adverse reactions were asthenia and nausea. Finally, patients reported an improvement in the quality of life (mean change in QLQ-C30 score), greater with talazoparib (3.0) vs. chemotherapy (−5.4), and the time to deterioration in the quality of life was significantly lengthened in the talazoparib arm. Recently, talazoparib obtained the marketing authorization for the treatment of locally advanced *BRCA1/2*-mutated HER2-negative mBCs. It has been approved for use as a monotherapy in patients who have already received treatment with an anthracycline and/or a taxane, as (neo) adjuvant therapy, for locally advanced or metastatic cancers, unless they were not eligible for this type of treatment. Patients with hormone receptor-positive BC must have previously received hormone therapy or be considered ineligible for hormone therapy to receive this form of treatment.

Rucaparib has been evaluated by Drew et al. [67] in a phase-II trial including 78 patients with *BRCA1/2*-mutated advanced breast or ovarian cancers. This trial had two cohorts, an oral treatment cohort and an intravenous treatment cohort. In each cohort, the first stage of the study included a dose-escalation phase. No objective response was observed in BC patients, both in the oral and intravenous cohorts. In contrast, 44% of patients in the intravenous cohort and 20% in the oral cohort exhibited disease stabilization over a 12-week period. Rucaparib was generally well tolerated. The Hoosier Oncology BRE09-146 group [68] reported a randomized phase-II trial evaluating rucaparib in combination with cisplatin in adjuvant therapy in TNBC or *BRCA1/2*-mutated BC with residual disease after neoadjuvant therapy (anthracyclines and/or taxanes). Patients were randomized (1:1) to receive cisplatin alone or in combination with rucaparib, with the main goal being two-year disease-free survival. A total of 128 patients were randomized, 22% of them carrying a *BRCA1/2* germline mutation. The toxicity profile was similar in both arms and the addition of rucaparib did not show significant improvement in two-year disease-free survival, regardless of *BRCA1/2* mutational status.

Sandhu et al. reported a phase-I trial evaluating niraparib in patients with sporadic advanced solid tumors or with a *BRCA1/2* mutation. The maximum tolerated dose was 300 mg of niraparib orally per day. Sixty patients were included in phase-I dose-escalation including 12 patients with mBC [20]. Among these patients, four carried a germline *BRCA1/2* mutation and 2/4 had a partial response to treatment. The toxicity was predominantly hematological with few grade 3 adverse effects. A phase-III randomized trial (the BRAVO trial, NCT01905592) is currently underway, comparing niraparib at a dose of 300 mg per day with chemotherapy of the physician’s choice in patients with locally advanced or metastatic HER2-negative BC, carrying a *BRCA1/2* germline mutation. Clinical trials on the use of PARPis in the treatment of mBC are summarized in Table 1.

### 3.2. Neoadjuvant Setting

The I-SPY 2 trial [70] was one of the first trials to evaluate the PARPi veliparib as a neoadjuvant treatment for localized BC. This trial included patients with stage II or III BC who had never received cytotoxic treatment for their BC. A total of 72 patients were randomized to receive veliparib-carboplatin in addition to standard neoadjuvant chemotherapy (weekly paclitaxel followed by doxorubicin plus cyclophosphamide), and 44 received standard therapy. Among the patients treated with a PARPi, 17% of them carried a germline *BRCA1/2* mutation. The addition of the veliparib-carboplatin combination to the standard neoadjuvant chemotherapy regimen allowed a doubling of the complete response rate in the TNBC subtype (51% vs. 26%). Another trial, confirming the effectiveness of PARPis in the neoadjuvant setting was the BrighTNess trial [71], which included women with stage II or III TNBC with or without a *BRCA1/2* germline mutation. Patients were randomized (2:1:1) to receive one of the following three treatment regimens: either paclitaxel (80 mg/m2, weekly for a total of 12 doses) plus carboplatin (area under the curve (AUC) 6 every three weeks for a total of four cycles) and veliparib (50 mg orally twice daily), either paclitaxel plus carboplatin and a placebo of veliparib or paclitaxel plus a placebo of carboplatin and a placebo of veliparib. After the weekly paclitaxel sequence, all patients received doxorubicin-cyclophosphamide chemotherapy every two or three weeks. A total of 634 patients were randomized to one of the three arms of the study, among them 15% carried a *BRCA1/2* germline mutation. Overall, the histologic complete response rate was significantly increased in the carboplatin-veliparib paclitaxel arm compared to the paclitaxel alone arm (53% vs. 31%).

In contrast, the addition of veliparib to the carboplatin-paclitaxel combination did not increase the proportion of patients achieving a complete tumor response. This trial corroborates the previous results on the benefit of carboplatin as a neoadjuvant treatment in TNBC. However, the presence of a *BRCA1/2* germline mutation did not appear to be associated with a greater benefit from the use of platinum and/or veliparib.

Furthermore, the GeparOLA study evaluated neoadjuvant olaparib in patients with non-metastatic HER2-negative T2-T4 or T1c BCs with lymph node involvement with either a *BRCA1/2* mutation (germline or somatic) or a high HRD score. Patients were randomized to receive chemotherapy combining weekly paclitaxel 80 mg/m2 and olaparib 100 mg twice daily or a weekly combination of paclitaxel 80 mg/m2 and carboplatin AUC 2, for 12 weeks. All patients received chemotherapy with epirubicin/cyclophosphamide thereafter. A total of 107 patients were randomized, 72% of which had TNBCs and 60% of patients had a *BRCA1/2* mutation. The histologic complete response rates were 55% with olaparib and 48% with carboplatin; however, the study could not rule out a 55% histological complete response rate in the olaparib arm, which was the primary outcome, but the results seemed more favorable in the subgroup of patients aged over 40 years or with hormone receptor-positive BC [72].

Regarding the use of talazoparib in this setting, Litton et al. [73] reported the results of a pilot study evaluating talazoparib as a neoadjuvant monotherapy in the treatment of localized (stage I to III) HER2-negative *BRCA1/2*-mutated BCs. Twenty patients (including 15 with a TNBC) were randomized to receive talazoparib at a dose of 1 mg/day for six months. The main objective was the histological complete response rate at six months, assessed on the surgical specimen. A total of 19 patients completed the six months of treatment and ten of them (53%) achieved a complete tumor response. The main toxicity was found to be hematological with grade I to III anemia leading to a need for dose reduction in nine patients and to erythrocyte transfusions in eight patients. The most commonly reported non-hematological adverse reaction was nausea, and all of these toxicities were easily managed with appropriate supportive care. Despite its small patients sample, this trial is the first to demonstrate the effectiveness of targeted therapy in the neoadjuvant setting in patients with *BRCA1/2* mutations without the addition of chemotherapy.

### 3.3. Adjuvant Setting

OlympiA is a phase-III, randomized, double-blind, placebo-controlled trial that evaluated olaparib as an adjuvant monotherapy in localized, HER2-negative *BRCA1/2*-mutated BCs [74]. The recruited patients had to have completed local treatment and adjuvant or neoadjuvant chemotherapy. Eligible patients should have had a TNBC (pT2 or pN1 for patients operated on straight away or residual disease for those receiving neoadjuvant chemotherapy) or expressing hormone receptors and should be without *HER2* gene amplification, pN2 for patients treated with adjuvant chemotherapy or high-risk residual disease after neoadjuvant chemotherapy, as defined by the CSP + EG score (clinical stage and post-treatment pathological stage incorporating estrogen receptor status and tumor grade). They were randomized in a 1:1 ratio to receive 300 mg olaparib twice daily or a placebo for 12 months. The main goal was invasive disease-free survival. Among the high-risk early BC patients with germline *BRCA1/2* mutations, 1 year of adjuvant olaparib was found to significantly reduce the risk of recurrence and prevent progression to metastasis (85.9% in the olaparib group and 77.1% in the placebo) [75]. Clinical trials on the use of PARPis in the neoadjuvant/adjuvant treatment of BC are summarized in Table 2.

## 4. Combination Strategies with PARPis in BC Treatment

### 4.1. PARPis and Chemotherapy

The mechanism of action of the majority of chemotherapeutic drugs is the damage to the DNA of cancer cells. PARPis alter DNA repair mechanisms and may be used as chemo-sensitizers. This hypothesis was examined by the trials cited above, as the BrighTNess, which combined veliparib with carboplatin and paclitaxel, or the GeparOLA, which combined olaparib and paclitaxel. However, despite obtaining promising results at the preclinical data analysis stage, the concomitant combination of a PARPi with a cytotoxic agent, including temozolomide, platinum salts, gemcitabine, or topoisomerase inhibitors, has been shown to be tricky in terms of toxicities, with an increase of hematological toxicity, leading to control by PARPi or chemotherapy dose reduction [76]. Regarding the effectiveness, the benefit of these combinations still remains to be established in populations with *BRCA1/2* or HRD mutations as well as in patients without such anomalies.

Veliparib (ABT-888) has primarily been evaluated in combination with platinum salt chemotherapy in advanced mBC. Indeed, its low activity in terms of “trapping” of PARP-1 allows for its development in combination with chemotherapy. Several phase-I trials have been reported evaluating the maximum tolerated dose and the potential efficacy of veliparib in solid tumors, alone [77] or in combination with other systemic treatments [78]. In 2017, Han et al. [79] reported the results of the BROCADE trial, a randomized phase-II trial evaluating the combination of veliparib with carboplatin and paclitaxel in the treatment of locally advanced or *BRCA1/2*-mutated HER2-negative BC. A third arm studied the combination of veliparib with temozolomide, an alkylating agent, that has demonstrated potential synergistic efficacy in combination with PARPis [80]. In each arm, veliparib was administered at low doses and both intermittently and concomitantly with the administration of chemotherapy (carboplatin or temozolomide). No significant difference was observed in the mean PFS with the addition of veliparib to carboplatin/paclitaxel. In contrast, there was a significant increase in the ORR in the experimental arms (ORR 77.8% vs. 61.3%, *p* = 0.027). The ORR of 61.3% in the carboplatin and paclitaxel arm confirmed previous data on the efficacy of platinum salts in the treatment of *BRCA1/2* mutated cancers [81]. As already described for the other PARPis, hematological adverse events such as neutropenia and thrombocytopenia were the most reported. Moreover, disappointing results were reported in the veliparib plus temozolomide arm with an ORR of 28.6%. In 2020, Dieras et al. [69] reported the results of the BROCADE3 trial, a randomized (2:1) phase-III double-blind trial, evaluating veliparib vs. placebo in combination with carboplatin (administered every 3 weeks) and paclitaxel (administered weekly) in the treatment of locally advanced or metastatic HER2-negative BC with a *BRCA1/2* germline mutation. Veliparib was administered orally at a low dose (120 mg twice daily) from day 2 to day 5, carboplatin with AUC 6 on day 1 every three weeks and paclitaxel on a weekly basis. If chemotherapy was discontinued, veliparib was continued as a full-dose maintenance monotherapy (300–400 mg/day). A crossover was planned for progression to veliparib monotherapy in patients receiving a placebo. The addition of veliparib showed an increase in the median PFS from 13.5 to 19.3 months after Blinded Central Review.

### 4.2. PARPis and Immunotherapy

Checkpoint inhibitors, such as anti-CTLA4 and anti-PD-1/PD-L1, seem to be more effective against cancer with a high mutagenic burden, probably because they have an increased propensity to produce neo-antigens for immune activations [82]. Therefore, tumors with *BRCA1/2* mutations or BRCAness, given the potentially high mutagenic burden, might be particularly responsive to checkpoint inhibitors [83].

Moreover, preclinical studies demonstrated an increased expression of immunologic markers such as tumor-infiltrating lymphocytes (TILs), especially in *BRCA1/2*-mutated TNBC [84]. In addition, in this specific subtype, olaparib has been shown to stimulate PD-L1 expression in tumor cells, improving anti-PD-1 antibody efficacy. Considering these data, clinical trials are underway to assess the combination of PARPis and immunological checkpoint inhibitors [85]. MEDIOLA is a phase-II trial evaluating the combination of olaparib plus durvalumab (anti-PD-L1 monoclonal antibody) in mBC HER2-negative with a *BRCA1/2* germline mutation. Patients were randomized to receive olaparib as monotherapy at a dose of 300 mg, twice daily for four weeks, followed by durvalumab 1500 mg intravenously for four-week cycles, with disease assessment every eight weeks. Domchek et al. published the results concerning the first 25 patients who showed a disease control rate at 24 weeks of nearly 50% without differences, according to hormonal or mutational status, and without an increase in toxicities during dual therapy [86]. MEDIOLA is the first study to report encouraging results for hormone receptor (HR)-positive germline *BRCA1/2*-mutated mBC treated with PARPis in combination with immune checkpoint inhibitors. Similarly, TOPACIO [87] is a phase-I/II trial evaluating the combination of niraparib with pembrolizumab (anti-PD-1 monoclonal antibody), particularly in metastatic TNBCs and in ovarian cancers. A total of 55 women were included in the BC cohort to receive niraparib at the recommended dose in phase I, i.e., 200 mg per day, in combination with pembrolizumab (200 mg intravenous on day 1/21). Of the 47 evaluable patients, 21% presented an ORR with five patients in complete response and five in partial response. Among 15 patients with a *BRCA1/2* germline mutation, 32% showed an ORR.

### 4.3. Other Innovative Combinations

Recent preclinical studies are interested in combining PARPis with other molecular targeted therapies to interfere with oncogenic pathways such as VEGF, IGF, PI3K and EGFR. For instance, the PI3K/mTOR signaling pathway is essential, particularly in detecting DNA DSBs, and it might be involved in the expression of BRCA1 and BRCA2 [76]. Therefore, PI3K inhibition could potentially weaken the HRR mechanism, resulting in a “BRCAness” tumor phenotype, regardless of *BRCA1/2* mutational status, increasing the effect of PARPis. Accordingly, early-phase clinical trials have been initiated evaluating the combination of PI3K or mTOR inhibitors with PARPis. In addition, several studies are currently underway evaluating treatments targeting molecules involved in cell cycle regulation or DNA repair, in association with PARPis [88]. These trials included a phase-II study of olaparib and ATR inhibitor (NCT02264678), a phase-IB study of olaparib and WEE1 inhibitor (NCT02511795), and a phase-II study randomizing olaparib as monotherapy vs. olaparib and WEE1 or ATR inhibitor in TNBC (VIOLETTE test, NCT03330847). A deeper knowledge of the biology of the HR-positive mBC with germline *BRCA1/2* mutations is necessary to define further studies with PARPis and immune checkpoint inhibitors, based on known lower response to anti-PD-L1 drugs and the potential sensitivity to CDK4/6 inhibitors; further studeis are needed to investigate the combination or the sequence of PARP inhibitors and CDK4/6 inhibitors (e.g., olaparib, fulvestrant and palbociclib–NCT03685331). Ongoing clinical trials of PARPis in combination with molecular targeted therapies are summarized in Table 3.

## 5. Acquired Resistance to PARPis

As PARPi therapy entered the clinical practice, resistance mechanisms to the treatment have emerged. Mutations in *BRCA1* and *BRCA2* genes are excellent targets for PARPis, but it has been shown that in 40–70% of patients the therapy is not effective [61].

The resistance to PARPis can be innate, when PARPis are ineffective from the beginning of the treatment for the presence of intrinsic resistance mechanisms, or acquired, when PARPis become ineffective after an initial benefit for the patient [89].

While innate resistance to PARPis is poorly known, several mechanisms of acquired resistance have been observed during the treatment with PARPis.

### 5.1. Restoration of BRCA1 and BRCA2 Functionality

Among the resistance mechanisms occurring during the treatment with PARPis, the restoration of BRCA1 and BRCA2 functionality by reversion mutations is the most common one [60,90,91,92,93,94,95,96,97,98,99].

Quigley et al. demonstrated the acquisition of a multi-nucleotide deletion that removes pathogenic mutation in *BRCA2* gene, restoring the correct open reading frame (ORF). Thus after some months of treatment, the recovered protein brings tumor cells to the loss of sensitivity to the PARPi Talazoparib [100]. Another group identified a deleterious germline mutation in *BRCA2*, whose carrier patient was treated with carboplatin and Rucaparib. After therapies, they identified in cfDNA twelve new somatic mutations that have occurred. Six new variants determined the restoration of the correct ORF [101]. Secondary restoration mutations have been observed also in other genes involved in the HRR system, for example in *RAD51B* and *RAD51C* [102].

### 5.2. Hypomorphic Forms of BRCA1

Another resistance mechanism observed during PARPi treatment is the partial restoration of HRR through increased activity of hypomorphic mutant BRCA1/2 proteins [103,104]. In particular, it has been observed that the knockout of 53BP1, a protein involved in DSB repair, can cause partial rescuing of the HRR in hypomorphic BRCA1 cells making them resistant to PARPi treatment [105].

### 5.3. Epigenetic Changes in HRR Genes

PARPi resistance can be the result of epigenetic changes in HRR genes. In particular, promoter hypermethylation of genes such as *BRCA1/2* determines a reduced expression of the corresponding mRNAs that results in HRD and PARPi sensitivity. On the opposite, the demethylation of these genes is associated with the restoration of protein expression and resistance to PARPi treatment [102,106,107].

### 5.4. Loss of End Resection Regulation

Another player involved in the PARPi resistance mechanism is the 53BP1 protein, whose loss in mice with a *BRCA1* mutation determines resistance to PARPi treatment restoring the HRR system. Furthermore, this mechanism seems to be dependent on ATM, another possible target in PARPi-resistant tumors [108]. PARPi resistance can also occur in presence of *BRCA1* mutations disrupting the N-term RING domain, taking advantage of the residual DNA repair activity of the protein [109,110].

Moreover, another player involved in the PARPi resistance mechanism is the PTEN protein, since the concomitant presence of PTEN loss and *BRCA1* mutation rewires the functionality of the HRR system [111].

### 5.5. Restoration of PARP-1 Activity

The PARP-1 enzyme is another player in PARPi resistance mechanisms, since mutations in the Zinc Finger Domain (ZFD) of PARP-1 that abolish DNA binding cause PARPi resistance [112]. Previous studies have demonstrated that a decrease in PARP-1 expression during PARPi treatment can promote the onset of a resistance mechanism [113]. In a different study, phosphorylation of PARP-1 by receptor tyrosine kinase c-Met has been shown to determine an increase in its enzymatic activity and reduce the binding to PARPi, thereby rendering cancer cells resistant to the treatment [114].

Another important player is the poly(ADP-ribose glycohydrolase (PARG), an enzyme the degrades the ADP-ribose chain synthetized by PARP-1. The depletion of PARG leads to the partial restoration of PARP-1 activity, inducing less sensitivity to PARPis [115].

### 5.6. NHEJ Suppression

Different repair pathways act during the cell cycle in the repair of DNA damage: NHEJ and HRR are complementary arms of the same system. When DNA strand breaks occur, a precise balance between these two regulatory systems is maintained by competition in binding broken strand ends, by Ku complex or MRN complex, respectively. The miR-662 can induce resistance to PARPis and platinum in *BRCA1*-mutant cells by targeting the Ku complex and restoring HRR. Indeed, in *BRCA1*-mutated ovarian tumors, the overexpression of miR-622 is associated with a reduced response to PARPi and platinum therapy [116,117].

### 5.7. Replication Fork Protection

Multiple studies showed that HRD tumor cells can develop PARPi resistance through the protection of the replication forks during DNA replication [118,119]. In particular, BRCA1/2-deficient cells have been shown to be able of reducing the recruitment of nucleases, such as MRE11 and MUS81, to the stalled forks, becoming resistant to the PARPi treatment without restoring the HRR [120,121].

### 5.8. Drug Concentration

The *ABCB1* gene encodes a transmembrane transporter P-glycoprotein that pumps out from cells a wide range of xenobiotic compounds, including drugs such as PARPis [122,123]. The overexpression of *ABCB1* has been associated with PARPi resistance [124]. P-glycoprotein inhibitors are able to restore the PARPi sensitivity in BRCA1-deficient cells [125].

## 6. Conclusions

Going forward, it has been well established that PARPis should be a component of the therapeutic strategy for BC arising in *BRCA1/2* mutation carriers. Furthermore, their application will likely move beyond metastatic setting to the adjuvant and neoadjuvant settings. In both OlympiAD and EMBRACA trials, PARPis demonstrated survival and quality of life benefit compared to chemotherapy. However, the side effects associated with platinum salts are known and are considered clinically significant. The PARPis talazoparib and olaparib as maintenance therapy, after initial cytotoxic chemotherapy (with or without platinum), have not been clearly evaluated, although patients with stable disease after chemotherapy may be included in EMBRACA. On the other hand, this strategy has been clearly provided in the BROCADE3 trial and may have played a major role in the PFS benefit observed in this trial.

Nowadays, both talazoparib and olaparib are registered in the treatment of metastatic or locally advanced *BRCA1/2*-mutated HER2-negative BC. However, patients must have received prior treatment with anthracyclines and taxanes in an adjuvant, neoadjuvant or metastatic setting. Moreover, PARPis might be used from the first line of cytotoxic treatment, but in tumors expressing hormone receptors, prior hormone therapy must have been administered or the patients must not be candidates for it. At this point, it is noteworthy to discuss different clinical strategies: in TNBCs with a *BRCA1/2* germline mutation and PD-L1 expression, which strategy should be adopted between taxanes-atezolizumab combination and PARPis? The OS benefit, in the IMpassion 130 study, favoring the chemo-immunotherapy combination does not seem to be impacted by the *BRCA1/2* mutational status [82,126]. On the other hand, in hormone receptor-positive BC patients, who have already been exposed to hormone therapy but not in combination with CDK4/6 inhibitors, should PARPis be preferred over a combination of hormone therapy plus cell cycle inhibitors? Given the increased OS associated with the latter combination [83,127,128], this is probably the most reasonable option. In the absence of visceral crisis, PARPis might be useful when hormone therapy is no longer effective. The is no direct comparison beteween PARPis and platinum salt-based chemotherapy: the efficacy of platinum salts in germline *BRCA1/2*-mutated patients is already underscored in the metastatic setting [129]. Finally, reinforcing the role of already approved PARPis, in a meta-analysis, Schettini et al. showed that PARPi regimens are correlated with an overall reduction in the instantaneous risk of progression of 41%, and about 14% reduction in the instantaneous risk of death. In addition, based on the results of subgroup analysis, they found an association between PARPis and PFS in ovarian cancer, prostate cancer, pancreatic cancer, melanoma and small-cell lung cancer, but also a statistically significant PFS improvement in BC, as it was already described in olaparib and talazoparib pivotal trial [130].

## Figures and Tables

**Table 1:** Clinical trials with PARP inhibitors in the treatment of mBC.

Trial	Study Design	No. of Pts	Phase	Patients’ Population	Primary Endpoint	Results	Approval
OlympiAD [63]	Olaparib vs. PCT	302	III	Advanced/metastatic gBRCA ≤2 prior lines	PFS	7.0 vs. 4.2 months	FDA/EMA approved
BROCADE3 [69]	CP + veliparib/placebo vs. Temozolomide + veliparib	337	II	Metastatic gBRCA ≤2 prior lines	PFS	14.1 vs. 12.3 months	-
EMBRACA [21]	Talazoparib vs. PCT	431	III	Advanced/metastatic gBRCA ≤3 prior lines	PFS	8.6 vs. 5.6 months	FDA/EMA approved

Abbreviations: C (carboplatin); P (paclitaxel); gBRCA (germline *BRCA1/2* mutation); PFS (progression free-survival); PCT (physician’s choice therapy).

**Table 2:** Clinical trials with PARP inhibitors in the neoadjuvant/adjuvant treatment of BC.

Trial	Study Design	No. of Pts	Phase	Patients’ Population	Primary Endpoint	Results
OlympiA [75]	Olaparib vs. placebo	1836	III	Early-stage gBRCA adjuvant therapy	IDFS	85.9% vs. 77.1%
BrighTNess [71]	CP + veliparib or CP + placebo or P + placebo + placebo→AC	634	III	Stage II or III TNBC gBRCA neoadjuvant therapy	pCR	58% vs. 53% vs. 31%
I-SPY 2 [70]	CP + veliparib/placebo→AC	116	II	Stage II or III TNBC neoadjuvant therapy	pCR	51% vs. 26%

Abbreviations: AC (doxorubicin plus cyclophosphamide); C (carboplatin); P (paclitaxel); gBRCA (germline *BRCA1/2* mutation); IDFS (invasive disease-free survival); pCR (pathological complete response).

**Table 3:** Selected ongoing trials with PARP inhibitors in combinations with molecular targeted therapies.

Clinical Trial Identifier	Study Design	Intervention/s	Setting	Primary Endpoint	Phase	Status
NCT02264678	330 Participants, Interventional, Parallel Assignment, open-label, Non-Randomized, Multi-center	Olaparib + Ceralasertib	Advanced solid malignancies not considered appropriate for further standard treatment	AE and SAE	I	Recruiting
NCT02511795	128 Participants, Interventional, Parallel Assignment, open-label, Non-randomized	Olaparib + Adavosertib	Refractory solid tumor	DLT, MTD, TEAEs	Ib	Completed
NCT03330847 (VIOLETTE)	273 Participants Interventional, Parallel Assignment, open-label, Randomized, Multi-center	Olaparib + Ceralasertib Olaparib + Adavosertib	Second or third line	PFS, ORR, DoR	II	Active, Not Recruiting
NCT03685331 (HOPE)	54 Participants, Interventional, Sequential Assignment, open-label, Non randomized	Olaparib + Palbociclib + Fulvestrant	First, second and third line	PFS	I/II	Recruiting
NCT01905592 (BRAVO)	215 Participants, Interventional, Parallel Assignment, open-label, Randomized, Multi-center	Niraparib	First, second line and third line	PFS	III	Active, Not recruiting

Abbreviations: Adverse Events, AE; Dose Limiting Toxicity, DLT; Duration of response, DoR; Maximum Tolerated Dose, MTD; Objective Response Rate, ORR; Progression Free Survival, PFS; Serious Adverse Events, SAEs; Treatment-emergent adverse events, TEAEs. The information was extracted from www.clinicaltrials.gov (accessed on 15 July 2021).
